# The Effects of Midline Cerebellar rTMS on Human Pharyngeal Cortical Activity in the Intact Swallowing Motor System

**DOI:** 10.1007/s12311-020-01191-x

**Published:** 2020-09-26

**Authors:** Ayodele Sasegbon, Nikola Niziolek, Mengqing Zhang, Craig J Smith, Philip M Bath, John Rothwell, Shaheen Hamdy

**Affiliations:** 1 Gastrointestinal (GI) Sciences, Division of Diabetes, Endocrinology and Gastroenterology, School of Medical Sciences, Salford Royal Hospital (part of the Manchester Academic Health Sciences Center (MAHSC)), University of Manchester, Salford, UK; 2 Manchester Centre for Clinical Neurosciences, Division of Cardiovascular Sciences, Lydia Becker Institute of Immunology and Inflammation, Salford Royal Hospital, Manchester Academic Health Sciences Centre (MAHSC), University of Manchester, Salford, UK; 3 Stroke Trials Unit, Division of Clinical Neuroscience, University of Nottingham, Nottingham, UK; 4 Stroke Medicine, Nottingham University Hospitals NHS Trust, Nottingham, UK; 5 Sobell Department of Motor Neuroscience and Movement Disorders, University College London, London, UK

**Keywords:** rTMS, Vermis, Midline, Swallowing, Dysphagia, Pharyngeal

## Abstract

We sought to compare the effects of 10 Hz cerebellar vermis (vs. unilateral hemispheric and sham) repetitive transcranial magnetic stimulation (rTMS) on cortical neuroelectrical activity and thereafter 10 Hz cerebellar vermis (vs. sham) rTMS on swallowing behaviour. Healthy participants (*n* = 25) were randomly allocated to receive vermis, unilateral hemisphere or sham 10 Hz cerebellar rTMS. Recordings were made using pharyngeal electromyography and manometry catheters, obtaining motor-evoked potentials (MEPs) and pressure recordings. The amplitudes of MEPs elicited using single-pulse TMS delivered to the pharyngeal areas of the motor cortex bilaterally were measured pre- and post-cerebellar stimulation. As in previous studies, abductor policis brevis (APB) MEPs were measured to assess post-rTMS modulation specificity. Swallowing was assessed using a swallowing accuracy task. Measurements were made at baseline and 15-min intervals for an hour post-intervention. Measurements involved TMS being used to elicit 10 MEPs bilaterally over the pharyngeal areas of the motor cortex, over the APB cortical representation adjacent to the pharyngeal area with the lowest resting motor threshold and 5 MEPs bilaterally over pharyngeal areas of the cerebellar hemispheres. Swallowing accuracy was assessed by giving participants 10 attempts to swallow and hit a digital target. Cerebellar vermis rTMS caused significant suppression of cortical pharyngeal MEP amplitudes compared with unilateral rTMS and sham (*P* = 0.0005, 0.002). APB and cerebellar MEP amplitudes were unaffected as were pharyngeal and APB MEP latencies. Following cerebellar vermis rTMS there was a significant reduction in swallowing accuracy compared with sham (*P* = 0.001). Our findings demonstrate cerebellar vermis rTMS exerts a suppressive effect on pharyngeal motor cortical activity and swallowing behaviour.

## Introduction

Our understanding of the neuronal circuitry within the central nervous system (CNS) which controls the sequence of muscular contractions required for a safe swallow has steadily progressed over the past nine decades.

Classically, the importance of the CNS in the control of swallowing was highlighted by the observation of dysphagia which occurred following strokes and other forms of pathology affecting the brain [1]. Subsequently, neurophysiological studies attempted to probe the relationships between different brain areas during the process of a normal swallow. Early studies predominantly focused on swallowing sensory and motor nuclei that exist within the brain stem. It is now known that these interconnected sensory and motor relays, collectively termed the central pattern generator (CPG), control the involuntary phase of swallowing [2]. However, the initiation of a swallow is a voluntary event and as such was not fully explained by the newly discovered ‘swallowing centres’ within the brainstem. Later studies attempted to address this gap in understanding by exploring the relationship between higher cortical centres and the CPG.

Previous invasive studies utilising microelectrodes embedded into the brains of animals and humans have been used to study the cortical representation of oral and pharyngeal musculature [3]. These studies were predominantly performed on anaesthetised animals. By applying targeted stimulation to cortical brain areas representing muscles involved with swallowing, it was possible to induce full swallowing responses [4–11]. These findings were later replicated following the focal stimulation of the same cortical regions in humans [3]. With these studies providing a foundation of knowledge, TMS has more recently been utilised by various groups to investigate swallowing in animals [12]. Subsequently, TMS was used to map out and investigate the cortical representation of swallowing musculature in humans [13]. In 1996, Hamdy et al. utilised single-pulse transcranial magnetic stimulation (TMS), a non-invasive method of examining targeted neuronal pathways, to map the hemispheric motor cortical representations of the muscles in the head and neck involved in swallowing [13]. During this study, the motor cortical ‘swallowing centres’ were observed to be asymmetrically active with one side being more active (or ‘dominant) than the other. Subsequent functional neuroimaging studies confirmed the occurrence of bilateral but commonly lateralised cortical activity during deglutition [13]. However, understanding of the role the cerebellum plays in swallowing has been slower.

In 1927, early animal studies by Mussen et al. [14] in anaesthetised cats first suggested that the cerebellum played a role in the control of swallowing. The electrical stimulation of the cerebellar vermis resulted in observable chewing and swallowing motions. Following this, in 1973 and 1975, studies by Reis et al. and Martner et al. confirmed earlier findings by demonstrating electrical stimulation of the cerebellar fastigial nuclei in alert cats resulted in changes to feeding behaviour [15, 16]. It would take several decades before functional imaging studies confirmed that the cerebellum was activated during deglutition. Interestingly, in contrast with the earlier animal studies where cerebellar vermis structures were electrically stimulated, functional imaging studies showed activity occurring over the cerebellar hemispheres [17, 18]. Additionally, several studies showed evidence in keeping with asymmetrical cerebellar hemispheric activation, with the left cerebellar hemisphere appearing to be more active than the right [19, 20]. Although the significance of this observed asymmetry is currently unclear, it is similar to what has been demonstrated to occur within the motor cortex.

Repetitive transcranial magnetic stimulation (rTMS) just like single-pulse TMS is an electromagnetic technique that can be used to target neuronal structures within the brain [21]. However, unlike single-pulse TMS, rTMS involves pulsing electromagnetic energy over the brain at a variety of different frequencies so as to influence subsequent neuronal activity [21]. High-frequency (5 Hz) rTMS applied over cortical pharyngeal motor areas has been shown to increase neuronal excitation within pharyngeal areas of the motor cortices while continuous low-frequency rTMS (1 Hz) over the same areas has been observed to cause neuronal suppression (a cortical ‘virtual lesion’) [22, 23].

In 2011, Jayasekeran et al. demonstrated that single-pulse TMS delivered over the pharyngeal motor areas of the cerebellar hemispheres and the cerebellar vermis was able to elicit pharyngeal motor-evoked potentials (PMEPs) detectable by an electromyography (EMG) catheter within the pharynx [24]. In the initial phase of this study, single-pulse TMS was applied to the pharyngeal motor areas of the cerebellar hemispheres and cerebellar vermis to identify the locations which reliably produced PMEPs of the greatest amplitudes. These three locations (right and left hemispheric cerebellar pharyngeal areas and cerebellar vermis) would serve as rTMS targets for stimulation. Within the study, PMEPs elicited by applying single-pulse TMS to the cerebellar vermis were of lower amplitudes than PMEPs elicited by applying single-pulse TMS to either of the cerebellar hemispheric pharyngeal motor areas [24]. Subsequently, in 2015, Vasant et al. performed a cerebellar rTMS study investigating the pharyngeal motor cortical effects of different frequencies of cerebellar rTMS when applied over the pharyngeal motor areas of the cerebellar hemispheres [25]. As in the earlier study by Jayasekeran, vermis-targeted single-pulse TMS elicited PMEPs of lower amplitudes than single-pulse TMS applied over the right or left cerebellar hemispheric pharyngeal motor areas [25].

To date, three rTMS studies have been performed examining the effects of cerebellar rTMS on the swallowing motor system. The first was performed by Vasant et al. and as described earlier, investigated the effects of different frequencies of unilateral hemispheric rTMS on motor cortical activity [25]. Ten hertz was discovered to be the optimum cerebellar excitatory rTMS frequency (in contrast with 5 Hz over the cortex). The second, a study by Sasegbon et al. successfully demonstrated the ability of unilateral hemispheric cerebellar rTMS to reverse the focal suppressive PMEP and behavioural effects in a hemispheric stroke model (cortical virtual lesion) [26]. Most recently, in 2020, Sasegbon et al. compared the effects of uni-hemispheric and bi-hemispheric cerebellar rTMS on cortical excitability and in reversing the negative effects of a cortical virtual lesion [27]. Bi-hemispheric cerebellar rTMS was observed to be more effective than unilateral cerebellar rTMS in its ability to increase PMEP amplitudes and its reversal effect.

At present, no study within the pharyngeal motor swallowing system has investigated the pharyngeal motor cortical or swallowing behavioural effects of rTMS targeted at the pharyngeal motor area over the cerebellar vermis. Therefore, to address this question, we aimed to compare the effect of cerebellar vermis (vs. unilateral hemispheric and sham) cerebellar rTMS on cortical neuroelectrical activity and thereafter cerebellar vermis (vs. sham) rTMS on swallowing behaviour in the unperturbed brain. Our hypothesis, based on the findings from earlier single-pulse cerebellar studies, was cerebellar rTMS would exert a similar excitatory effect to unilateral hemispheric cerebellar rTMS. This would potentially indicate the lack of need for precise cerebellar rTMS targeting when administering stimulation.

## Method

The study was designed as a single-blinded, double-protocolled healthy participant study. The first protocol contained an active 10 Hz unilateral hemispheric cerebellar rTMS arm, an active 10 Hz vermis cerebellar rTMS arm and a midline (vermis) sham cerebellar rTMS arm. The second protocol contained vermis active and midline sham 10 Hz cerebellar rTMS arms. The first study protocol aimed to assess pharyngeal motor cortical excitability, measured in terms of MEP amplitudes, following unilateral and vermis cerebellar rTMS. The second protocol aimed to assess swallowing behaviour, measured as swallowing accuracy, following vermis cerebellar rTMS.

**Fig. 1:**
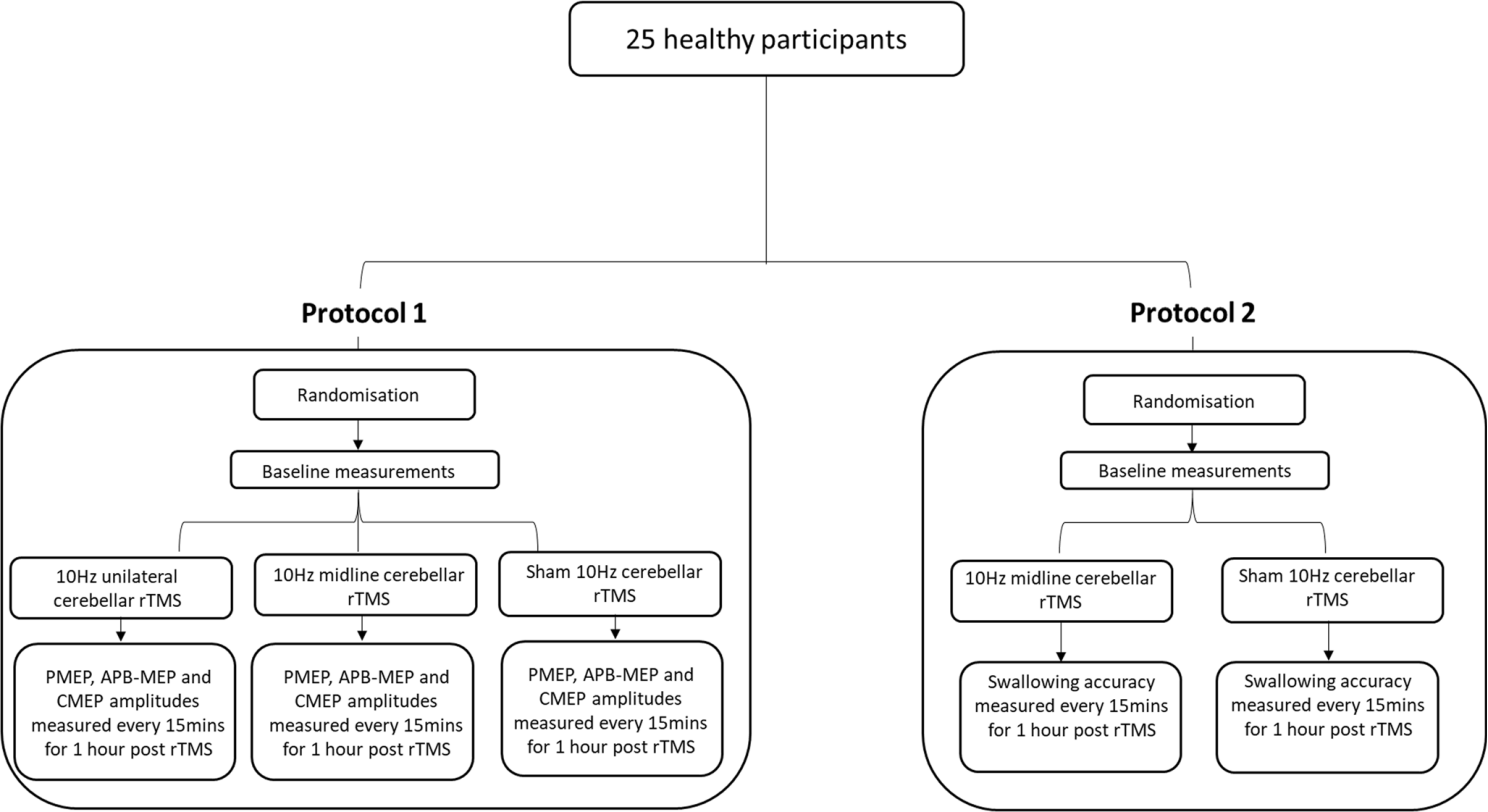
Study flow diagram

Over the course of the study, each participant was expected to participate in up to five study sessions, receiving active (unilateral hemispheric and vermis) and sham cerebellar rTMS (Fig. 1). Study sessions were a minimum of 48 h apart. The first study protocol was completed prior to the second being completed. Participants in each protocol were blinded and pseudorandomly allocated to receive active or sham rTMS.

The North West NHS research ethics committee (19/NW/0119) assessed and granted ethical approval for the study. All laboratory study sessions took place in the gastrointestinal laboratories at Salford Royal Hospital NHS Foundation Trust.

### Outcome Measures

Primary outcome measures were PMEP amplitude (as a marker of motor cortical activity) and swallowing accuracy (as a marker of swallowing behaviour), see Fig. 1.

Motor evoked potential (MEP) amplitudes have been shown by previous studies in the field to be correlated with changes in motor cortical activity [28].

Previously published studies by our group have demonstrated changes in swallowing accuracy (measured using a reaction targeting task) are correlated with changes in swallowing behaviour observed using videofluoroscopy (VFS) [29, 30].

Secondary outcome measures were MEP latencies and adverse events. Adverse events described in the literature as occurring following single-pulse TMS or rTMS include seizures and headaches [31]. These were assessed by observation and asking participants after each laboratory session to report any such events in the period after the experiment.

### Participant Recruitment

Based on the design and data from previous studies [26, 27, 32, 33], it was calculated that a minimum number of 12 participants would be required per study arm in protocol 1 and 10 participants per study arm in protocol 2 in order to obtain a statistical power of 80% (*P* = 0.05) with an effect size of 40%.

Over the duration of the study, 25 healthy participants were formally recruited resulting in 13 participants in protocol 1 and 12 in protocol 2. Exclusion criteria included the following: epilepsy, use of anxiolytics or antidepressants, lack of capacity to consent, implanted metal in the head, the presence of an implanted medical electrical device, pregnancy and the existence of a previous history of dysphagia. Participants were given a minimum of 24 h to read through the participant information sheet before being asked to give written consent for participation in the study. Eleven women and 4 men participated in protocol 1 with a median age of 22 ± 3 years. Eight women and 6 men participated in protocol 2 with a median age of 23 ± 5 years.

### Electromyography

Pharyngeal electromyography (EMG) recordings were performed using intraluminal catheters. Catheters were 3.2 mm in diameter (Gaeltec Ltd, Isle of Skye, UK) and contained two ring electrodes from which recordings were made. The electrodes were comprised of the non-ferrous metal platinum so as to prevent electromagnetic TMS pulses creating any distortion in recorded MEPs. Catheters were positioned at depths of 13–15 cm within the pharynx, as measured from the ring electrodes and secured in place using medical tape. Insertion was performed trans-orally or trans-nasally (according to participant preference). This was in keeping with the method of anatomical pharyngeal EMG catheter placement described by previous studies [30, 33–35]. In order to minimise electrical interference, a skin electrode (H69P, Tyco Healthcare, Gosport, UK) was placed on each participant’s sternocleidomastoid and connected to earth.

Abductor policis brevis (APB) EMG recordings were performed by attaching two skin electrodes (H69P, Tyco Healthcare, Gosport, UK) roughly 2 cm apart on the thenar eminence of the hand contralateral to a participant’s ‘dominant’ pharyngeal motor cortical hemisphere. To minimise distortion a third skin electrode was connected to each participant’s radial prominence and connected to earth. APB-MEP recordings were measured and analysed as an experimental control as has been described in previous studies [23].

EMG signals required amplification and cleaning prior to being analysed by the computer program Signalsoft (version 4.0, CED, Cambridge, UK). To that end, input leads were initially connected to a Cambridge Electronic Design (CED) 1902 preamplifier which was in turn connected to two electrical ‘noise’ cleaning devices connected in parallel (HumBug, Quest Scientific, North Vancouver, Canada). These were connected to a CED data interface (CED micro 1401, UK). Signals were then sent to a desktop computer (Dell, Gosport, UK) for offline analysis.

### Swallowing Reaction Time Task

An accurate swallow involves the prompt initiation of the full repertoire of neurological and muscular processes involved in performing a normal swallow while an inaccurate swallow can be caused by factors including slowed cortical initiation of a swallow or incoordinate pharyngeal muscular contractions [36]. Therefore, swallow accuracy with respect to hitting a predetermined temporal target can be used as a surrogate measure of swallowing function. This approach has been validated in several studies [26, 27]. Furthermore, VFS-based assessments of deglutition reveal alterations in swallowing that correlate with observed changes in swallowing accuracy [29].

Swallowing accuracy was assessed using a reaction time task performed as has been described in previously published studies by our group [23, 26]. In brief, a 1.5-mm catheter (Gaeltec Ltd, Isle of Skye, UK) containing a manometry sensor was inserted trans-nasally or trans-orally and positioned within the pharynx 13–15 cm from the nostrils or lips (as described in protocol 1). The catheter was attached to a custom-built ‘swallow timer’ which recorded timing data and measured changes in intraluminal pharyngeal pressure. Participants were prompted to swallow and ‘hit’ a predetermined target window (see protocol 2 below) generated by a swallowing timing program (Medical Physics, SRFT, UK) and displayed on the screen of a desktop computer (Dell, Berkshire, UK). The number of correct swallows out of 10 was recorded for subsequent analysis. Each swallow for all participants was facilitated with the use of water delivered in 3 ml aliquots via a 50-ml syringe connected to an orally placed plastic infusion tube.

### Single-Pulse Transcranial Magnetic Stimulation

Single-pulse TMS, used for evoking MEPs over targeted cortical pharyngeal, cortical APB and cerebellar pharyngeal motor areas, was performed using a 7-cm (in diameter) figure of eight electromagnetic coil attached to a Magstim 200 signal generator (The Magstim Company, Whitland, UK). The coil and stimulator capacitor generated a magnetic field with a strength of 2.2 Tesla which was predominantly focussed at the intersection between the two halves of the electromagnetic coil. Single-pulse TMS was delivered as has been described in previous studies [25, 33]. Briefly, the electromagnetic coil was held flat, pressed to the scalp with the anterior aspect of the coil inclined at an angle of 45° towards the sagittal plane. Due to the posterior location of the cerebellum, for cerebellar TMS, the electromagnetic coil was held upside down with its handle pointing superiorly before being pressed against the scalp.

For the purpose of cortical pharyngeal and APB motor hotspot identification, single-pulse TMS was used to evoke PMEPs and APB-MEPS from pharyngeal motor cortical representations over both cortical hemispheres and the APB motor cortical representation over the cortical hemisphere with the lowest pharyngeal resting motor threshold (RMT). This approach has been described in several previous studies [25, 26]. In order to achieve this, Magstim generator signal intensity was set at 40% of maximum and gradually increased until reproducible MEPs were evoked. For cerebellar pharyngeal area motor hotspot identification over the right and left cerebellar hemispheres and cerebellar vermis, the process was similar to that used for cortical mapping but stimulator intensity was commenced at 30%. The two starting TMS intensities for cortical and cerebellar motor area hotspot identification were chosen because pharyngeal and APB area RMTs published in previous studies [25, 26] are described as being above these values.

MEP amplitudes were measured using TMS pulses delivered at an intensity of 120% of the RMT of the hemispheric cortical or cerebellar areas being measured in keeping with previous studies [24–26]. No post-mapping measurements were made over the cerebellar vermis.

### Repetitive Transcranial Magnetic Stimulation

RTMS was administered over the cerebellum using a 7-cm in diameter figure of eight electromagnetic coil connected to a Magstim super-rapid stimulator (The Magstim Company, Whitland, UK). The maximal output of the system was 1.8 Tesla. Coil orientation and placement was performed in an identical manner as the method used for the delivery of single-pulse TMS. Active unilateral 10 Hz rTMS was administered over the right cerebellar hemispheric pharyngeal motor hotspot. Active 10 Hz rTMS was administered over the cerebellar vermis below the cranial inion at the location of the hotspot identified during single-pulse TMS motor area mapping. In almost all participants, this was equidistant between the marked locations of the right and left cerebellar pharyngeal motor areas (located during single-pulse TMS mapping). Two hundred and fifty pulses were administered at 10 Hz at an intensity of 80% of pharyngeal RMT capped at 90% of APB area RMT. These stimulation parameters have been used in previous studies in the field and are meant to ensure the safe delivery of rTMS to the cerebellum [25, 26]. Sham cerebellar rTMS was delivered by tilting the coil 90° from its flattened orientation and touching the edge of one of its wings to a participant’s scalp (over the cerebellar vermis). This served to provide an approximation of the experience of receiving rTMS by replicating the pressure on the scalp and the sound of the electromagnetic pulses as they travel through the coil.

### Magnetic Resonance Imaging and Neuronavigation

**Fig. 2:**
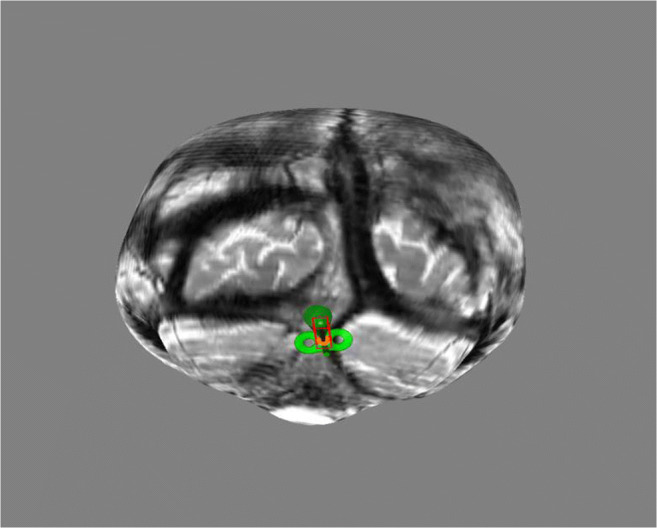
Cerebellar vermis stimulation point from a participant’s MRI using frameless functional MRI stereotaxy (Brainsight 2, Rogue Research, Montreal, Canada)

MRI-guided frameless stereotaxy (Brainsight 2, Rogue Research, Montreal, Canada) was used to co-register the TMS coil position over the cerebellar vermis in one participant as a means of confirming the accuracy of the anatomical neuronavigation approach used for the remaining participants (Fig. 2). The participant was selected on the basis of having had a previous MR image which could be used for frameless stereotaxy. The MRI scanner used was a 3-Tesla Phillips Intera-Achieva (Amsterdam, Netherlands). Subsequently, Brainsight neuronavigation software (Brainsight2, Rogue Research, Montreal, QC, Canada) on a standalone Apple iMac computer (Apple, Uxbridge, UK) was used during co-registration of MRI scans and positioning of the figure of eight coil [37, 38].

### Study Protocols

#### Protocol 1

As seen in Fig. 1, the PMEP study was completed first before the behavioural study was commenced. At the start of each study, participants were randomised to the active or sham rTMS arm using the computer program SPSS (IBM, Armonk, NY, USA). During a study session, each participant was asked to sit comfortably in a chair. Two skin electrodes were attached over their right thenar eminence with an earth electrode attached over their radial eminence. Another skin electrode was attached over the sternocleidomastoid over one side of their neck and earthed. A pharyngeal intraluminal EMG catheter was then measured and inserted trans-nasally or trans-orally into their pharynx as described previously. All skin electrodes and the pharyngeal catheter were then connected to the CED preamplifier.

To prepare for single-pulse TMS mapping, a disposable surgical cap was positioned over each participant’s head, tied in place and taped down. The nasion, inion and cranial vertex were identified and their positions marked on the cap. The location of the cranial vertex was determined by marking the midpoint along a line stretching from the inion to the nasion. Pulses of TMS were then administered over the left and right cortical motor areas. As each pulse was delivered, pharyngeal and APB EMG traces displayed on a computer screen were examined for the emergence of a MEP. If one was not observed, the coil was moved 1–2 cm in all directions and another pulse was delivered. This continued until a PMEP or APB-MEP was elicited. If no MEPs were observed to occur at a given TMS intensity, Magstim generator intensity was increased by 5% and the process was recommenced. When a PMEP or APB-MEP was detected the position of the coil was marked on the disposable cap and the sites evoking both responses were then interrogated to ensure quantifiable responses could be reliability elicited—these locations were designated the ‘hot-spots’. Using this approach, it was, therefore, possible to identify the pharyngeal motor cortical areas bilaterally and the APB motor area adjacent to the dominant pharyngeal motor hemisphere (defined as the hemisphere which evoked the largest PMEP with the lowest RMT). A similar approach was used to identify the pharyngeal motor representations over both cerebellar hemispheres and the cerebellar vermis [33]. For the purposes of cortical motor mapping, TMS intensity was commenced at 40% of maximal Magstim generator output and steadily increased until MEPs were observed. For cerebellar motor mapping, TMS intensity was commenced at 30% of the maximum Magstim generator output. RMTs were defined as the lowest TMS intensity required to evoke: cortical or cerebellar PMEPs ≥ 20 μV or cortical APB-MEPs ≥ 50 μV in amplitude in 5 out of 10 pulses. As per previous published studies, the more active or dominant pharyngeal cortical motor hemispheric area was defined as the hemisphere with the lowest RMT [13].

With the motor areas marked and the RMTs documented, baseline measurements were made by delivering 10 pulses of single-pulse TMS at 120% of RMT bilaterally over the pharyngeal and APB cortical motor locations. Additionally, 5 pulses of TMS were delivered over the cerebellar pharyngeal motor areas. Each set of measurements at each location generated MEPs detected by the pharyngeally positioned EMG catheter or APB electrodes. MEP amplitudes and latencies were assessed and averaged before being converted to percentage changes from individual baseline. During each set of measurements, participants were asked to avoid coughing, speaking or swallowing as much as possible.

Ten Hz active or sham cerebellar rTMS was then delivered as per each participant’s assigned group. Following rTMS, repeat measurement were made at 15-min intervals (0, 15, 30, 45 and 60 min) up to an hour post-cessation of rTMS.

#### Protocol 2

As in protocol 1, randomisation was performed, surgical caps were positioned, pharyngeal intraluminal catheters were inserted, TMS motor mapping performed and RMTs were established. Subsequently, the pharyngeal EMG catheter was removed and the manometry catheter was inserted. This was then connected to the swallowing timing system (Medical physics, Salford Royal NHS Trust, UK). The manometry catheter was then calibrated so the presence of a swallow could be accurately determined. Following this, the pressure threshold above which the system recognises a swallow as having occurred was set at ≈ 45% of maximum swallowing pressure, a value which has been used in previous manometric studies of swallowing accuracy as a point of reference, above which, pressure responses are felt as more stable [26]. Measurements of swallowing accuracy were then performed as described in previous studies [26, 33]. In brief, participants were prompted to perform 10 normal swallows. They were then prompted to complete 10 fast swallows. Swallowing was prompted visually via a cue on a computer screen (Dell, Berkshire, UK) in front of each participant and audibly via a loud clicking sound. Participants were cued to swallow once every 10 s. The differences between the normal and fast swallow latencies were used to calculate a graphical swallowing target that appeared on the computer screen as a time window. To obtain the primary outcome measure of swallowing accuracy, participants were prompted to swallow and try to hit the target window as many times out of 10 as possible.

After baseline swallowing accuracy was recorded, cerebellar active or sham rTMS was administered as per protocol 1. Repeat swallow performance measurements were then made at 15-min intervals (0, 15, 30, 45 and 60 min) for an hour.

### Data Analysis

#### Protocol 1

MEP amplitudes—measured as the distance between the highest peak of a MEP to its lowest trough in microvolts—and MEP latencies—measured as the time from the delivery of a TMS pulse to the onset of a MEP—were recorded at each targeted cortical and cerebellar motor location. Each group of 10 cortical recordings and 5 cerebellar recordings were subsequently averaged before being converted to percentage changes from individual baselines. Amplitude measurements were analysed using MEP analysis software (Signal version 4.0) installed on a personal computer (Dell, Berkshire, UK).

In keeping with the method of analysis utilised in previous neurophysiological studies in this field [25, 33], pharyngeal PMEP amplitudes over the right and left cortical hemispheres and pharyngeal MEP amplitudes over the right and left cerebellar hemispheres were analysed to see if their percentage changes from baseline were significantly different from one another. If no significant difference was found, the percentage changes from baseline were combined and averaged before being analysed. Interventions (unilateral, vermis and sham rTMS) were compared with each other and with baseline using repeated-measures analysis of variance (rmANOVA). Subsequently, post hoc one-way ANOVAs were performed after Bonferroni correction. Study data were analysed using SPSS Statistics v. 22.0 (IBM Corp., Armonk, NY, USA).

#### Protocol 2

In protocol 2, swallowing accuracy was measured as the number of prompted swallows on target out of a total of 10. These values were then converted into percentage changes from individual baseline before being analysed using rmANOVA as per protocol 1.

All data are reported as mean ± standard error of the mean unless stated otherwise. A *p* value of < 0.05 was taken to be indicative of significance.

## Results

There were no observed or reported adverse events following the use of single-pulse cortical TMS, single-pulse hemispheric cerebellar rTMS or vermis cerebellar rTMS.

### Hemispheric Cortical and Cerebellar Motor Hotspots

**Fig. 3:**
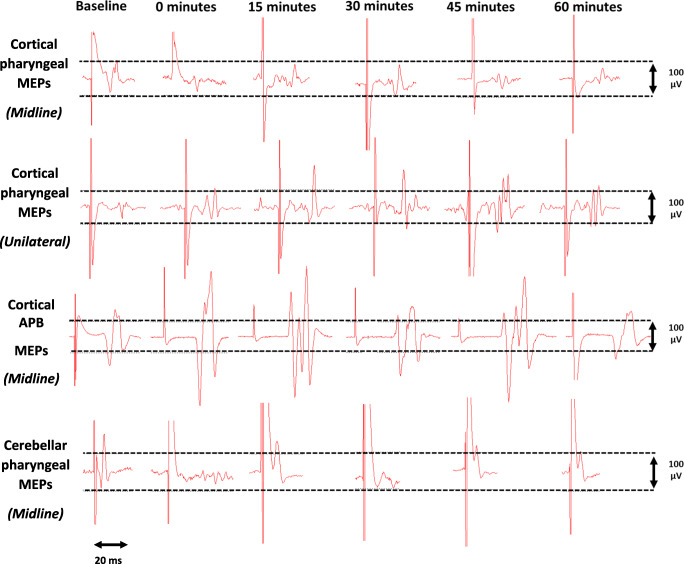
Sample cortical PMEP, APB-MEP and cerebellar PMEP traces measured from the dominant pharyngeal cortical hemisphere and the right cerebellar hemisphere pre and post cerebellar rTMS. Midline indicates MEP traces measured pre and post vermis cerebellar rTMS. Unilateral indicates traces pre and post unilateral cerebellar rTMS

Targeted single-pulse TMS consistently evoked detectable PMEPs, APB-MEPs and C-PMEPs from uni-hemispheric cortical and cerebellar motor representations as can be seen in Fig. 3. RMTs for combined hemispheric cortical pharyngeal areas, cortical APB motor areas and combined cerebellar pharyngeal motor areas for protocols 1 and 2 are depicted in Table 1. Baseline PMEP amplitude and latency data can be seen in Tables 2 and 3.

**Table 1:** Median cortical and cerebellar resting motor thresholds (RMT) for protocols 1 and 2

	Vermis cerebellar rTMS	Unilateral cerebellar rTMS	Sham cerebellar rTMS
**Protocol 1**			
‘Dominant’ cortical hemisphere	73 ± 6.0	75 ± 9.0	75 ± 5.9
‘Non-dominant’ cortical hemisphere	80 ± 4.5	85 ± 7.5	80 ± 7.5
APB	45 ± 6.5	45 ± 6.5	44.5 ± 9.3
Cerebellar hemispheres (combined)	55 ± 5.0	55 ± 5.0	55 ± 2.5
**Protocol 2**			
Dominantcortical hemisphere	70 ± 5.0	–	70 ± 5.0
Non-dominant cortical hemisphere	80 ± 5.0		80 ± 5.0
APB	45 ± 7.0		45 ± 7.0
Cerebellar hemispheres (combined)	50 ± 5.0		50 ± 5.0

**Table 2:** Baseline cortical pharyngeal, cortical APB and cerebellar pharyngeal MEP amplitudes in microvolts (μV) for protocol 1-averaged across individuals and hemispheres

	Vermis cerebellar rTMS	Unilateral cerebellar rTMS	Sham cerebellar rTMS
**Protocol 1**			
MEP amplitudes μV			
Cortical pharyngeal	55.7 ± 29.0	159.8 ± 154.5	58.4 ± 29.1
Cortical thenar	327.7 ± 143.1	2183.6 ± 1946.5	316.4 ± 87.3
Cerebellar pharyngeal	26.0 ± 5.3	213.6 ± 268.6	26.0 ± 6.4

**Table 3:** Cortical pharyngeal and APB and cerebellar pharyngeal MEP latencies in milliseconds (ms) for protocol 1

	Vermis cerebellar rTMS	Unilateral cerebellar rTMS	Sham cerebellar rTMS
**Protocol 1**			
**Cortical pharyngeal (combined) (ms)**			
Baseline	8.9 ± 0.6	9.1 ± 0.3	8.8 ± 0.7
0 min	8.9 ± 0.7	9.0 ± 0.7	8.9 ± 0.7
15 min	9.0 ± 0.7	9.4 ± 0.5	9.0 ± 0.7
30 min	9.1 ± 0.4	9.2 ± 0.8	9.1 ± 0.4
45 min	8.8 ± 0.6	8.8 ± 0.6	8.8 ± 0.6
60 min	8.8 ± 0.3	9.0 ± 0.4	8.8 ± 0.3
**Cortical APB (ms)**			
Baseline	22.2 ± 0.5	20.5 ± 1.2	22.1 ± 0.5
0 min	22.2 ± 0.7	20.3 ± 0.8	22.2 ± 0.6
15 min	22.2 ± 0.5	21.7 ± 1.0	21.9 ± 0.6
30 min	22.0 ± 0.7	21.5 ± 0.8	21.8 ± 1.6
45 min	22.4 ± 0.9	21.6 ± 1.0	22.2 ± 0.7
60 min	22.4 ± 0.6	22.0 ± 1.2	22.3 ± 0.7
**Cerebellar pharyngeal (combined) (ms)**			
Baseline	6.4 ± 0.7	5.8 ± 0.4	6.4 ± 0.7
0 min	7.0 ± 0.7	5.7 ± 0.5	7.0 ± 0.8
15 min	7.0 ± 0.6	5.5 ± 0.6	7.3 ± 0.5
30 min	6.5 ± 0.7	6.3 ± 0.8	6.9 ± 0.9
45 min	6.7 ± 0.9	5.8 ± 0.7	6.8 ± 0.7
60 min	7.0 ± 0.7	5.6 ± 0.6	7.0 ± 0.5

In protocol 1, 9 of the 12 participants studied had a dominant (more active) right cortical pharyngeal motor hemisphere in the active vermis arm and 9 of 13 in the active unilateral arm. In the sham arm, 10 of 12 had a dominant right cortical hemisphere. In protocol 2, 8 of 12 participants had a dominant right hemisphere in the active arm and 9 of 12 in the sham arm. No participant was observed to exhibit co-dominance (equal RMTs over both right and left cortical pharyngeal motor hemispheres). One participant in each protocol was observed to switch their dominant cortical pharyngeal motor hemisphere between study sessions.

#### Protocol 1

Over the right and left cortical hemispheres, using the cranial vertex as a reference point, the mean pharyngeal representation motor locations were 4.8 cm (standard deviation (SD) ± 1.2 cm) anterior (y coordinate) and 2.2 cm (SD ± 0.7 cm) lateral (x coordinate) and 4.9 cm (SD ± 0.9 cm) anterior and − 1.4 cm (SD ± 0.9 cm) lateral, respectively.

Over the right and left cerebellar hemispheres, using the cranial inion as a reference point, the mean pharyngeal motor locations were 6.2 cm (SD ± 2.4 cm) inferior (y coordinate) and 3.1 cm lateral (SD ± 1.1 cm) (x coordinate) and 6.2 cm (SD ± 2.4 cm) inferior and − 3.2 cm lateral (SD ± 0.8 cm), respectively.

#### Protocol 2

Over the right and left cortical hemispheres, using the cranial vertex as a reference point, the mean pharyngeal area motor locations were 4.9 cm (SD ± 0.7 cm) anterior (y coordinate) and 1.9 cm (SD ± 0.9 cm) lateral (x coordinate) and 4.8 cm (SD ± 0.6 cm) anterior and − 1.6 cm (SD ± 0.7 cm) lateral.

Over the right and left cerebellar hemispheres, using the cranial inion as a reference point, the mean pharyngeal area motor locations were 6.3 cm (SD ± 1.4 cm) inferior (y coordinate) and 2.2 cm lateral (SD ± 2.0 cm) (x coordinate) and 6.3 cm (SD ± 1.4 cm) inferior and − 2.8 cm lateral (SD ± 0.9 cm).

### Protocol 1: the Effects of Vermis Cerebellar rTMS on Hemispheric Cortical and Cerebellar Motor Activity in an Unperturbed System

Following cerebellar rTMS, rmANOVA revealed there was no significant difference in PMEP amplitudes between dominant and ‘non-dominant’ pharyngeal hemispheres (*F*_10, 165_ = 1.116, *P* = 0.353) or between right and left cerebellar hemispheres (*F*_10, 150_ = 1.015, *P* = 0.433). As a result, PMEP data from both cortical and cerebellar hemispheres were combined for further analysis. This was in keeping with the published methodology of previous studies in the field [26].

#### MEP Amplitudes

##### Cortical Pharyngeal

**Fig. 4:**
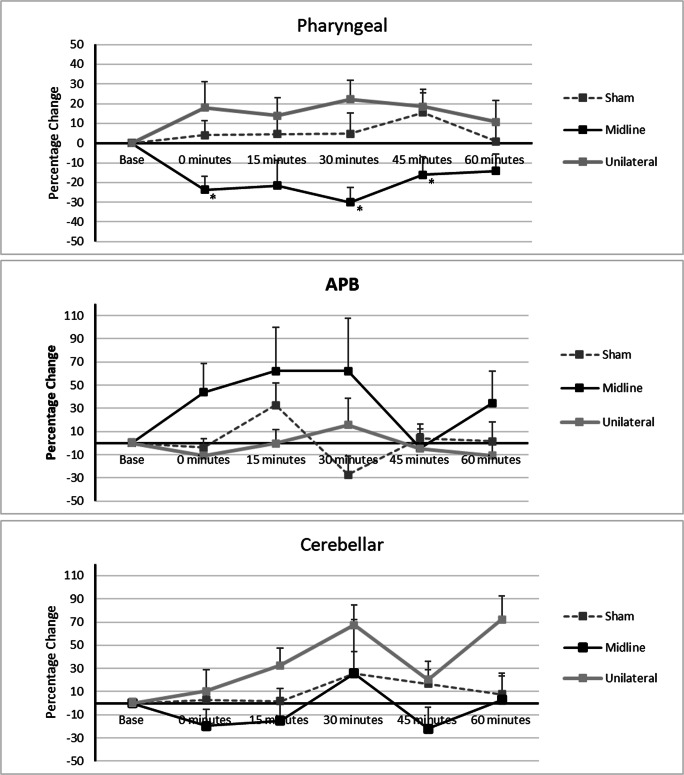
Graphs of PMEP amplitudes showing percentage changes from baseline with vermis and sham cerebellar rTMS. **a** Combined pharyngeal cortical area, **b** APB cortical area, and **c** cerebellar pharyngeal area. Asterisks indicate statistical differences between interventions (*P < 0.05; **P < 0.005). Error bars indicate standard error of the mean

RmANOVA revealed a significant time × intervention interaction for vermis, unilateral and sham cerebellar rTMS (*F*_13, 291_ = 2.331, *P* = 0.006) with a significant main effect for intervention *F*_3, 68_ = 10.031, *P* = 0.0005, but not for time *F*_4, 291_ = 0.789, *P* = 0.541. Post hoc one-way ANOVA of study data revealed vermis rTMS provoked a significant decrease in PMEP amplitudes compared with unilateral cerebellar rTMS and sham rTMS, *P* = 0.0005 and 0.002, respectively. Vermis cerebellar rTMS was significantly different to unilateral at 0, 30 and 45 min, (*F*_2, 2, 2_ = 4.6, 7.8, 4.2, *P* = 0.016, 0.001 and 0.038) and sham at 30 min (*F*_2_ = 7.8, *P* = 0.002) (Fig. 4).

##### Cortical Thenar

RmANOVA did not reveal a significant Time × Intervention interaction for mid-line, unilateral and sham cerebellar rTMS (*F*_10, 172_ = 1.8, *P* = 0.060).

##### Cerebellar Pharyngeal

RmANOVA did not reveal a significant Time × Intervention interaction for mid-line, unilateral and sham cerebellar rTMS (*F*_10, 142_ = 1.8, *P* = 0.061).

#### MEP Latencies

##### Cortical Pharyngeal

RmANOVA did not reveal a significant time × intervention interaction for mid-line, unilateral and sham cerebellar rTMS (*F*_12, 182_ = 1.1, *P* = 0.400).

##### Cortical Thenar

RmANOVA did not reveal a significant time × intervention interaction for mid-line, unilateral and sham cerebellar rTMS (*F*_11, 152_ = 1.1, *P* = 0.334).

##### Cerebellar Pharyngeal

RmANOVA did not reveal a significant time × intervention for mid-line, unilateral and sham cerebellar rTMS (*F*_7, 97_ = 0.6, *P* = 0.747).

### Protocol 2: the Swallowing Behavioural Effects of Vermis Cerebellar rTMS

**Fig. 5:**
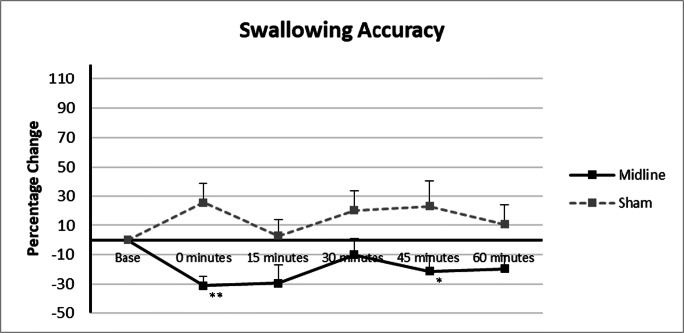
Graph of swallowing accuracy showing percentage changes from baseline with vermis and sham cerebellar rTMS. Asterisks indicate statistical differences between interventions (*P < 0.05; **P < 0.005). Error bars indicate standard error of the mean

RmANOVA of study data identified a significant time × intervention interaction for vermis vs. sham cerebellar rTMS (*F*_10, 225_ = 1.9, *P* = 0.048). There was a significant main effect of intervention (*F*_2, 45_ = 19.2, *P* = 0.0005) but not of time (*F*_5, 225_ = 1.1, *P* = 0.372). Post hoc one-way ANOVA showed a significant reduction in swallowing accuracy compared with sham (*P* = 0.001) (Fig. 5).

One-way ANOVA showed vermis cerebellar rTMS to be different to sham at 0 and 45 min (*F*_1, 1_ = 15.3, 5.8, *P* = 0.001 and 0.024, respectively).

## Discussion

Despite our initial hypothesis which stated that 10 Hz rTMS delivered over the cerebellar vermis will increase PMEP amplitudes in a manner similar to that seen with 10 Hz cerebellar hemispheric rTMS [25–27], we observed cerebellar vermis rTMS resulted in a decrease in PMEP amplitudes and disrupted swallowing accuracy. As such, these findings merit further discussion.

### Neurophysiological Effects

Early animal studies in primates and non-primates established the locations of the cerebral motor cortical representations of the muscles of the oral cavity and pharynx involved in the process of swallowing [4–11]. The importance of these regions to the performance of a normal swallow was bolstered by later studies that sought to selectively lesion regions of the cortex corresponding to muscles involved in mastication and swallowing [39, 40]. They showed that damage to these brain regions impaired muscular activity involved in swallowing [39, 40]. Later, human studies using invasive [3] and non-invasive means [13] are built upon this work and established the locations of oropharyngeal cortical motor areas in humans. The importance of these areas in the control and initiation of swallowing had been established for some decades with the observation that dysphagia often occurs following strokes [41]. In a similar manner to the somatotopic layout of the primary motor cortices, regions of the cerebellar surface also correspond to different body areas. The cerebellum has its own lesser-known, motor homunculus. However, unlike the cortex, the same muscle groups are represented multiple times in different locations over the cerebellum [42]. The cerebellar homunculus is multi-limbed [43] and can be described as spider like. This complexity may explain why motor maps of the cerebellum were published later than cortical motor maps.

In humans, most work has been done mapping the locations of the limbs on the cerebellar surface. For example, studies show the hands and feet are represented bilaterally in several distinct places, over the superior and inferior aspects of the cerebellum [44–46]. Fewer studies have attempted to formally map the motor representation of facial muscles. In 2001 and 2020, Grodd et al. and Boillat et al. found that the lips and tongue were represented bilaterally over the cerebellar hemispheres [43, 44]. In addition, the tongue was also represented over the cerebellar vermis. By contrast, information regarding the somatotopic locations of pharyngeal musculature is limited but is indirectly provided by functional imaging and TMS studies. It has thus been shown that both cerebellar hemispheres are active during the process of swallowing [19] and that PMEPs can be evoked by TMS targeted over the cerebellar hemispheres and vermis [24, 25] suggesting some degree of somatotopy. In this study, comparing the neuroelectric and functional effects of rTMS delivered to the cerebellar pharyngeal hemispheric sites with the vermis arguably increases our understanding of discrete cerebellar representations for the pharynx/swallowing. This is less comprehensive than a full intracerebellar microelectrode stimulation mapping study, but such a study is not possible in a human model.

Like the cerebrum, the cerebellum is composed of a cortex overlying deeper neuronal structures [42, 47]. Below the cortex are deep motor nuclei including the dentate and fastigial nuclei which can be found in the cerebellar hemispheres [47, 48]. RTMS stimulation of the cerebellum leads to changes within the cortex which communicates with motor nuclei including the dentate nuclei [47]. With respect to dentate nuclei, these in turn communicate with the thalamus before going on to modulate contralateral cortical motor areas [47]. This constitutes one of the potential pathways through which cerebellar rTMS may exert a cortical effect. Another potential pathway is via the cerebellar cortical modulation of fastigial nuclei. Fastigial nuclei synapse with neurones within the brain stem sensory and motor relay responsible for the control of swallowing known as the CPG [47, 48]. The CPG itself communicates with higher centres including the cerebral motor cortices [2]. However, due to the fact that deeper cerebellar motor nuclei lie within the cerebellar hemispheres, it is unclear as to precisely how their activity is modulated by rTMS targeted at the cerebellar vermis. Three peduncles physically attach the cerebellum to the brainstem and are the conduits through which it influences activity in the brainstem and more distantly in the motor cortices.

Our study shows high-frequency rTMS targeted over the cerebellar vermis in an unperturbed pharyngeal motor system, appears to exert a small but significant bilateral suppressive effect on cortical PMEP amplitudes compared with active unilateral and sham rTMS. This was seen to occur without causing significant changes in the amplitudes of APB cortical area or cerebellar pharyngeal area MEPs. Due to the novel nature of our findings and the dearth of studies in this area, no direct comparisons can be made between our data and the findings of previously published studies. The three studies that have been published to date exploring the effects of cerebellar rTMS in the pharyngeal motor system have only applied rTMS to the pharyngeal motor areas over the cerebellar hemispheres [25–27]. Their key findings can be summarised as: hemispheric cerebellar rTMS increases cortical PMEP amplitudes; 10 Hz rTMS appears to be the optimal frequency for prompting pharyngeal cortical area this excitation; hemispheric (10 Hz) cerebellar rTMS can reverse the suppressive PMEP and behavioural effects of a cortical virtual lesion; and bilateral hemispheric cerebellar rTMS is more excitatory than unilateral. No suppressive effect has been described previously.

Indirect comparisons can be made which put our study findings into context. Firstly, the 2015 cerebellar rTMS study by Vasant et al. [25] showed excitatory frequency specificity existed for hemispheric cerebellar rTMS. Multiple frequencies were studied including the following: 1 Hz (considered to be inhibitory when delivered over the pharyngeal motor cortex), 5 Hz (the optimum excitatory frequency for pharyngeal cortical areas), 10 Hz and 20 Hz. The only frequency which provoked a significant increase in cortical PMEP amplitude was 10 Hz. These study findings, when taken in combination with the earlier finding by Gow et al., that 5 Hz cortical rTMS causes optimal excitation [22], illustrate the existence of site and frequency-specific rTMS excitatory effects between the cortical and cerebellar hemispheres. This implies the existence of further as yet unreported excitatory differences in rTMS responsivity between brain areas. Our demonstration of a site-specific vermis rTMS suppressive PMEP effect, while novel, is perhaps not entirely unexpected when considered in that context.

Our study findings can be considered to be akin to the 2007 cortical rTMS study by Mistry et al. which established the now commonly used cortical virtual lesion hemispheric stroke model [23]. Low-frequency rTMS delivered over the dominant pharyngeal motor cortical representation was found to suppress subsequent PMEP amplitudes and disrupt swallowing behaviour [23]. Additionally, there are similarities in the extent of suppression observed by Mistry et al and in this study. In the Mistry study the maximal suppressive effect was ≈ 35% [23] compared with a maximal effect of 30% in this study. Despite the fact that it is well known that strokes affecting the cerebellum and brainstem can cause dysphagia, no ‘infratentorial virtual lesion’ stroke model exists. The suppressive PMEP effects of vermis 10 Hz cerebellar rTMS may constitute such a physiological model—albeit a model of unclear future clinical applicability. Interestingly, bilateral cortical suppressive effects were seen following vermis rTMS as opposed to uni-hemispheric suppression following a cortical virtual lesion. In the future, with refinement, it may emerge that within physiological experiments, vermis cerebellar rTMS is able to provoke greater suppressive cortical PMEP and disruptive swallowing behavioural effects than the existing hemispheric virtual lesion stroke model.

Our observation that vermis cerebellar rTMS did not appear to cause intrinsic cerebellar modulatory effects is similar to the findings of Vasant et al. and Sasegbon et al. [25, 26] which demonstrated that hemispheric cerebellar rTMS caused cortical excitatory changes without causing cerebellar excitatory changes.

### Behavioural Effects

Vermis cerebellar rTMS caused a significant disruption in swallowing behaviour, manifest as a decrease in swallowing accuracy. This supported the results of the PMEP study and given that disruptive swallowing accuracy changes have been correlated with observable changes on VFS [49], infers that vermis cerebellar rTMS is able to exert a physiologically detectable disruptive effect. In the study by Mistry et al., which developed the hemispheric cortical stroke virtual lesion [23], it was postulated that the suppressive behavioural effects of low-frequency rTMS over the dominant motor swallowing area were possibly caused by a disruption in the cortical initiation of swallowing. This translated into a reduction in swallowing accuracy. Due to the neuronal pathways which exist between the cerebellum and cortical motor areas [24, 50, 51], the reduction in swallowing accuracy seen post vermis cerebellar stimulation may be caused by a similar disruption in cortical initiation of volitional swallows. However, a more direct effect of vermis rTMS on deeper cerebellar motor nuclei [47] or brainstem regions involved with the control of swallowing such as the CPG [2] cannot be ruled out.

### Potential Mechanism of Action

Vermis cerebellar rTMS was observed to cause bi-hemispheric pharyngeal motor cortical area suppression; however, the precise pathways and mechanisms through which this occurs are currently incompletely understood. The cerebellum predominantly functions as a suppressive motor regulatory organ [52]. Increases in cerebellar outputs act to suppress motor cortical activity and vice versa [52]. As a result, vermis cerebellar rTMS may cause increased activity along cerebello-cortical neuronal pathways and a subsequent suppressive effect on pharyngeal area activity. This may be mediated by potential stimulation of deep cerebellar fastigial nulcei (which lie along the vermis of the cerebellum) compared with the more laterally located dentate cerebellar nuclei [42, 52, 53] Fastigial nuclei likely influence hemispheric motor areas via their connections to the CPG within the brainstem [48]. Unfortunately, no detailed cerebellar rTMS functional imaging studies have been conducted in the swallowing motor system.

In this context, it is important to note that while no significant difference was found between APB-MEP amplitudes post cerebellar vermis rTMS compared with sham or baseline, APB-MEPs showed a graphical appearance of increased amplitudes. This may be due to cerebellar vermis rTMS acting on cerebello-cortical pathways which may be more excitatory with respect to APB motor areas than with respect to pharyngeal motor areas.

### Potential Applicability

The suppressive PMEP and behavioural effects observed following vermis 10 Hz cerebellar rTMS map out the first steps along a path which may lead to the development of a robust cerebellar/brainstem stroke model which can be used alongside the existing hemispheric stroke model in future neurophysiological experiments.

At present, it is unclear where this technique may be applied in the field of neurostimulation as a means of addressing neurogenic dysphagia. Most therapeutic approaches aim to use neurostimulation to increase activity within targeted brain regions [35]. It is thought that this increase in neuronal excitability serves as the impetus to subsequent beneficial neuroplastic changes. However, an emerging concept within the field of neurophysiology is metaplasticity. It is thought that by preconditioning the brain with high or low-frequency rTMS prior to an rTMS intervention, one can reduce inter- and intra- participant response variability and provoke greater neuronal excitation or deeper suppression [54]. Although no studies on cerebello-cortical or within cerebellar metaplasticity exist in the swallowing motor system, a role for vermis rTMS may emerge as a pre-interventional conditioning stimulus.

Additionally, while we have shown 10 Hz rTMS over the cerebellar vermis is able to decrease cortical PMEP amplitudes in contrast with the increased amplitudes observed when it is applied over cerebellar hemispheric pharyngeal areas [25], no studies have investigated the effects of different frequencies of vermis targeted rTMS on motor cortical activity. Of particular interest would be 1 Hz rTMS which is known to cause reductions in motor cortical PMEP amplitudes when applied over cortical pharyngeal representations [23]. However, it is important to note that Vasant et al. did investigate the effects of different frequencies of hemispheric-targeted cerebellar rTMS on cortical PMEP amplitudes and found 1 Hz rTMS did not lead to any significant changes [25].

Another neuromodulatory approach that will need to be investigated in the cerebello-cortical motor swallowing system is theta-burst stimulation (TBS). TBS is a newer variant of traditional rTMS wherein pulses are delivered at frequencies much higher than those utilised in rTMS [55]. Intermittent TBS applied over the cerebellar hemispheres has been shown to cause reduced motor cortical MEP amplitudes while continuous TBS has been shown to cause the opposite [55]. Finally, transcranial alternating current stimulation (TACS) applied over the cerebellar hemispheres has also been shown to be able to modulate motor cortical activity [56]. However, cerebellar TACS has not been used within the pharyngeal motor swallowing system. Furthermore, no studies have applied TACS to the cerebellar vermis.

### Limitations

Despite statistically significant findings, the study possessed some limitations. Firstly, it is difficult, without more detailed brain mapping with neuroimaging, to determine if vermis cerebellar rTMS is acting directly on cerebellar neurons or on deeper motor nuclei within the cerebellum or brainstem. As such, the observed effects may not arise entirely from neuronal modulation within the cerebellum and there is a possibility the observed effects may be as a result of changes to brainstem structures. However, in support of the cerebellar origin of the observed effects is the fact that cerebellar PMEPs seen in this study and others wherein single-pulse TMS applied to pharyngeal motor areas of the cerebellum (over the hemispheres or vermis) have elicited PMEPs with a similar morphology and latency to cortical PMEPs [24, 25]. The presumption has been that these represent cerebellar PMEPs; however, this has not been definitively confirmed. In addition, a 1995 study by Ugawa et al. found that brainstem responses could only be evoked reliably using a double cone coil (instead of the figure of eight coil used for this study).

Secondly, the coil tilt technique was used to perform sham cerebellar rTMS stimulation [57]. While this can approximate the scalp pressure and the sound made by rTMS, it is not a perfect sham. However, it is also true to say that as of yet no perfect sham intervention exists in rTMS as such a technique would also have to mimic the scalp sensation participants experience when electromagnetic pulses penetrate their heads. The coil tilt technique has also been used in multiple published studies [23, 33].

## Conclusion

We have shown that in contrast with the excitation provoked by hemispheric cerebellar rTMS, vermis cerebellar rTMS causes transient pharyngeal motor cortical area suppression and disruption of swallowing behaviour and suggests site specificity in the cerebellum’s role in modulating swallowing. Future studies of differential suppressive or excitatory effects of different rTMS frequencies over the cerebellar vermis combined with detailed functional brain imaging will further refine our understanding of the underlying neurophysiological effects of cerebellar rTMS.
